# A Case of Drug‐Induced Hypersensitivity Syndrome Caused by Apalutamide

**DOI:** 10.1002/iju5.70085

**Published:** 2025-08-15

**Authors:** Yuki Tanaka, Yui Fujimura, Koya Morishita, Yuta Kashiwagi, Satoshi Katsuno, Tatsuya Nagai

**Affiliations:** ^1^ Okazaki City Hospital Okazaki Aichi Japan

**Keywords:** apalutamide, DIHS, prostate cancer

## Abstract

**Introduction:**

Apalutamide, an androgen receptor antagonist for prostate cancer, rarely causes drug‐induced hypersensitivity syndrome (DIHS).

**Case Presentation:**

A 75‐year‐old male with prostate cancer and multiple bone metastases developed grade 2 rash and grade 3 liver dysfunction according to the Common Terminology Criteria for Adverse Events (CTCAE) 3 weeks after starting apalutamide with a GnRH antagonist, followed by a 3‐day fever. Ten days later, symptoms worsened to grade 3 rash and grade 4 liver dysfunction. He met five diagnostic criteria for DIHS. Hormonal therapy was discontinued, and prednisolone plus intravenous immunoglobulin (IVIG) was administered. Fatigue resolved within 2 days, rash by day 6, and liver function improved to grade 2 by day 10. The patient is currently on abiraterone and a GnRH antagonist without adverse events.

**Conclusion:**

This report highlights the importance of caution and regular blood tests when using apalutamide owing to the risk of DIHS.

**Abbreviations**
ADT: androgen deprivation therapy
AEs: adverse events
ALT: alanine aminotransferase
AST: aspartate aminotransferase
CT: computed tomography
DIHS: drug‐induced hypersensitivity syndrome
GnRH: gonadotropin‐releasing hormone
HHV‐6: human herpes virus 6
IVIG: intravenous immunoglobulin
mCSPC: metastatic castration sensitive prostate cancer
OS: overall survival

**Summary**

- Drug‐induced hypersensitivity syndrome (DIHS) is rarely caused by apalutamide.
- Treatment of DIHS involves the use of prednisolone; however, if it is ineffective, the addition of intravenous immunoglobulin can sometimes lead to recovery.
- Japanese patients should be cautious of severe drug eruptions, including DIHS, when using apalutamide.

## 1 Introduction

Apalutamide, combined with androgen deprivation therapy (ADT), improves overall survival (OS) metastatic castration‐sensitive prostate cancer (mCSPC), compared to placebo [1].

Drug‐induced hypersensitivity syndrome (DIHS) is a severe rash characterized by fever and multiorgan involvement, and is often associated with drug allergy and human herpes virus 6 (HHV‐6) reactivation. The mortality rate is as high as 10%. Early diagnosis and therapeutic intervention are crucial for effective treatment [2].

DIHS is a rare adverse event associated with the use of apalutamide. This case report describes a patient in whom DIHS caused by apalutamide was effectively treated with a combination of prednisolone and intravenous immunoglobulins (IVIG).

## 2 Case Report

**FIGURE 1:**
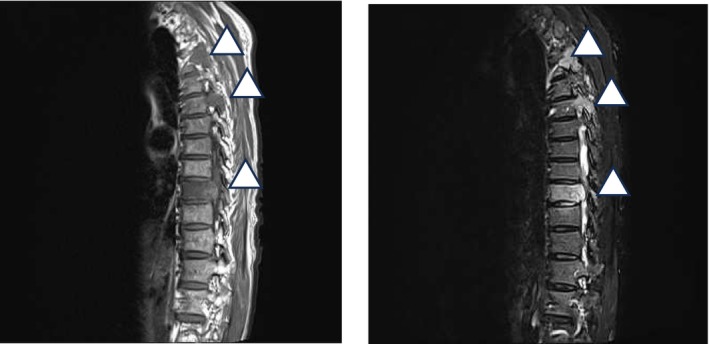
Magnetic resonance imaging reveals metastatic bone tumors at the levels of Th3, Th5, and Th9, compressing the spinal cord.

A 75‐year‐old Japanese male (height, 154 cm; weight, 39 kg) with no history of allergies was urgently transported to the emergency department because of bilateral lower limb weakness and bladder and bowel dysfunctions. Computed tomography (CT) revealed pathological bone fractures (Figure 1). Decompression and fixation surgeries were performed in the Orthopedic Department. The prostate‐specific antigen level measured at that time was 822 ng/mL, and transrectal prostate biopsy revealed prostate cancer with a Gleason score of 4 + 4. Treatment with apalutamide 240 mg/day and gonadotropin‐releasing hormone (GnRH) receptor antagonist was initiated.

**FIGURE 2:**
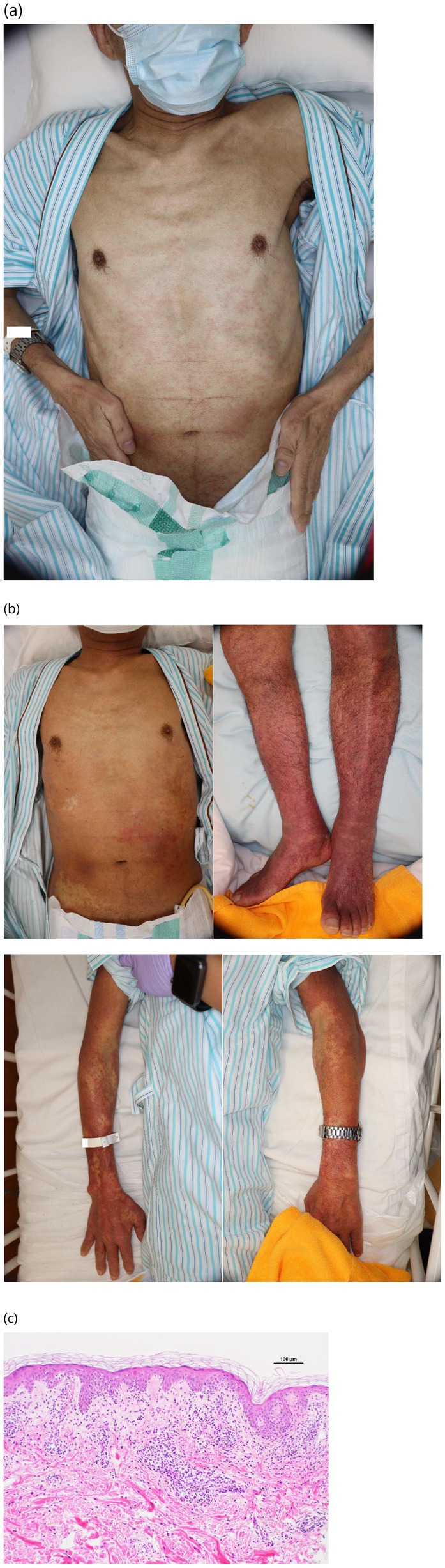
(a) A widespread erythema is observed from the chest to the abdomen. (b) Worsening erythema extending from the chest to the abdomen. Erythema was also observed in the limbs. (c) Lymphocyte exocytosis in the epidermis and inflammatory cell infiltrates, predominantly composed of lymphocytes in the dermis.

**FIGURE 3:**
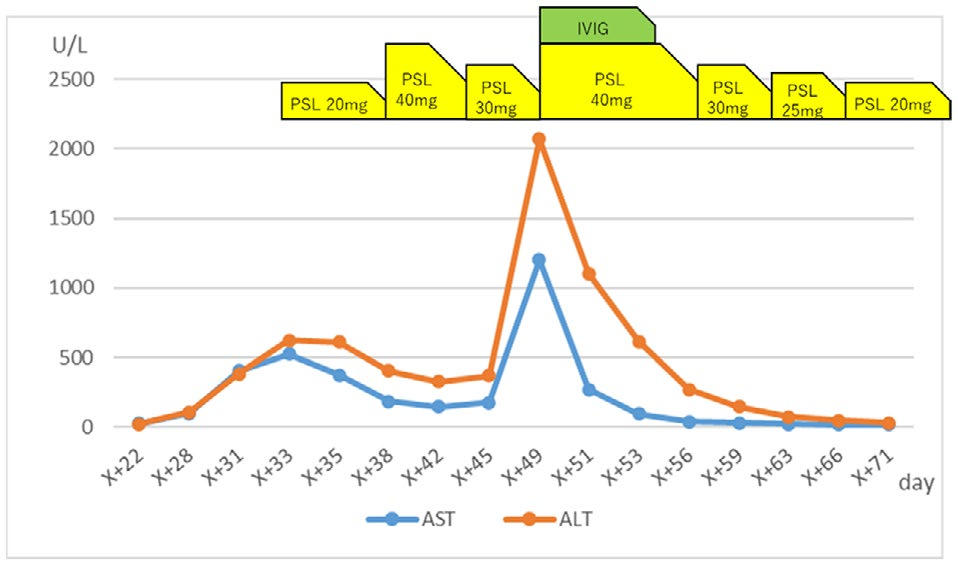
The changes in AST and ALT levels along with the medications used. IVIG was administered for 5 days after the day when a significant elevation in liver function was detected. Subsequently, as liver function and rash showed signs of improvement, prednisolone was gradually tapered. AST, aspartate aminotransferase; ALT, alanine aminotransferase; IVIG, intravenous immunoglobulin; PSL, prednisolone.

Three weeks after initiating hormone therapy, a rash was observed (Figure 2a). Blood tests revealed aspartate aminotransferase (AST) and alanine aminotransferase (ALT) levels of 101 and 109 U/L, respectively, and atypical lymphocytes (Figure 3). Apalutamide was discontinued, and the patient was referred to the dermatology department. The initial dermatological diagnosis was grade 2 drug eruption. Three days later, the patient developed drug‐induced fever and was prescribed oral prednisolone (20 mg daily).

One week later, the patient experienced worsening skin symptoms (grade 3 skin rash) (Figure 2b). After skin biopsy, the prednisolone dose was increased to 40 mg/day. However, 8 days later, the patient complained of fatigue, and blood tests revealed grade 4 liver dysfunction (AST, 1201 U/L; ALT, 2069 U/L; total bilirubin, 2.5 mg/dL). Therefore, intravenous immunoglobulin (IVIG) therapy was initiated because DIHS that occurred during apalutamide treatment was suspected. After 5 days of IVIG treatment, liver function gradually improved. Subsequently, the prednisolone dose was gradually reduced, and the patient was discharged when the dose reached 20 mg/day.

Skin biopsy revealed lymphocyte exocytosis in the epidermis, leading to a diagnosis of drug eruption (Figure 2c). The drug‐induced lymphocyte stimulation test (DLST) for apalutamide was negative. However, reactivation of HHV‐6 was confirmed after discharge. Given the absence of other suspected drugs, a definitive diagnosis of DIHS was established. The patient is currently being treated with abiraterone and GnRH receptor antagonists. Six months after discharge, there were no signs of prostate cancer progression or DIHS recurrence on blood test results or CT images, and the patient's prostate‐specific antigen level was 0.033 ng/mL.

## 3 Discussion

Prostate cancer is the most common cancer in men. Recently, new hormonal therapies, including apalutamide, an androgen receptor inhibitor have emerged. The TITAN trial demonstrated that apalutamide + ADT showed a significant prolonged of OS in mCSPC [1]. However, apalutamide causes adverse events (AEs), particularly rash (29.2% in TITAN; 53.6% in Japanese patients) [3] Severe drug rashes from apalutamide are rare, and DIHS is even less common, with no prior case reports.

DIHS incidence is 1.5 cases per 100 000 person‐years [4]. Similar to Stevens–Johnson syndrome and toxic epidermal necrolysis, DIHS is characterized by fever, lymphadenopathy, liver dysfunction, and a generalized rash. HHV‐6 reactivation distinguishes DIHS from the other two conditions. Although typical drug eruptions improve after discontinuing the suspected drug, DIHS symptoms often worsen. Diagnostic criteria for DIHS [5] (Figure 3) exist; however, diagnosis is challenging as symptoms typically develop 2–6 weeks postmedication, and evaluation items gradually become positive. In this case, six criteria were ultimately met, leading to a diagnosis of atypical DIHS. Despite a negative DLST result for apalutamide, DIHS was diagnosed because DLST is not essential for the diagnosis. No other new medications were introduced, and no DIHS recurrence occurred with continued prior medications (excluding apalutamide) (Table 1).

**TABLE 1:** The diagnostic criteria for DIHS. Typical DIHS must meet all the criteria, whereas atypical DIHS must meet criteria 1 through 5.

Criteria
1. Erythema: Occurs with delayed onset after the administration of particular drugs and rapidly enlarges, often developing into erythroderma.
2. Duration: Persists for ≧ 2 weeks, even after discontinuation of the causative drug.
3. Fever: A fever ≧ 38°C.
4. Liver dysfunction
5. Hematological abnormalities (One or more of the following)
a. Leukocytosis (≥ 11 000/mm^3^)
b. Appearance of atypical lymphocytes (≥ 5%)
c. Eosinophilia (≥ 1500/mm^3^)
6. Lymph node enlargement
7. Reactivation of HHV‐6
Typical DIHS: All of 1–7 must be present.
Atypical DIHS: All of 1–5 must be present but 4 may be substituted by other severe organ damage.

Abbreviations: DIHS, drug‐induced hypersensitivity syndrome; HHV‐6, human herpes virus 6.

The treatment of DIHS involves discontinuation of the causative drug and aggressive supportive care. It has been suggested that earlier drug discontinuation results in a better prognosis [6]. As for pharmacological treatment, systemic steroid administration is widely accepted. The standard treatment starts with prednisolone at 0.5–1 mg/kg/day; however, in severe cases, 1 mg/kg/day is recommended [7, 8]. If the disease cannot be controlled with adequate steroid treatment, high‐dose immunoglobulin therapy can be considered. In Japan, immunoglobulins are administered at 0.4 g/kg/day for 2–5 days.

The exact cause of DIHS remains unknown. However, since DIHS is a severe drug eruption and considering that Japanese individuals are more prone to drug rashes with apalutamide, Japanese patients should be monitored for AEs such as DIHS. In one study, low body weight was identified as a risk factor for cutaneous AEs caused by apalutamide in Japanese individuals [9]. Since patients with low body weight are more likely to develop skin rashes, it is essential to distinguish between mild drug eruptions and severe drug reactions such as DIHS. Moreover, according to Zachary, in patients weighing less than 67 kg, when apalutamide was administered at a dose of ≤ 180 mg, the incidence of rash decreased, and CRPC‐free survival was comparable to that of a group receiving 240 mg of apalutamide [10]. It is thus important to carefully consider the initial dose of apalutamide in patients with low body weight. When a skin rash appears, clinicians should evaluate the patient's systemic condition, including fever, liver dysfunction, and complete blood counts, considering the possibility of a severe drug eruption. Early recognition of severe cutaneous eruptions is crucial. The primary physician should instruct the patient to promptly seek medical attention if a rash appears and collaborate with a dermatologist to appropriately manage the rash when it occurs.

No known cross‐reactions exist between apalutamide and other androgen receptor antagonists. Therefore, the selection of a subsequent treatment for apalutamide‐induced DIHS is challenging. In this case, abiraterone was used as the next‐line therapy, assuming that a different mechanism of action would reduce the likelihood of cross‐reactions.

## 4 Conclusion

Herein, we present a rare case of apalutamide‐induced DIHS. It is important to note the risk of severe drug eruptions, including DIHS, when apalutamide is administered to Japanese patients with prostate cancer and low body weight.

## Consent

Informed consent was obtained from the patient for the publication of this case.

## Conflicts of Interest

The authors declare no conflicts of interest.
